# 1567. Perspectives of People with HIV (PWH) 6 Months Following a Switch to Cabotegravir and Rilpivirine Long-acting (CAB+RPV LA) in an Observational Real-world US Study (BEYOND)

**DOI:** 10.1093/ofid/ofad500.1402

**Published:** 2023-11-27

**Authors:** Dima Dandachi, Douglas Cunningham, William M Valenti, John Phoenix, Kaitlin Nguyen, Paula Teichner, Ashley Jean-Louis, Maria Reynolds, David Richardson, Cindy Garris

**Affiliations:** University of Missouri - Columbia, Columbia, Missouri; Pueblo Family Physicians, Phoenix, Arizona; Trillium Health, Rochester, New York; Huntridge Family Clinic, Las Vegas, Nevada; ViiV Healthcare, RTP, North Carolina; ViiV Healthcare, RTP, North Carolina; RTI-Health Solutions, Research Triangle Park, North Carolina; RTI Health Solutions, Research Triangle Park, North Carolina; RTI Health Solutions, Research Triangle Park, North Carolina; ViiV Healthcare, RTP, North Carolina

## Abstract

**Background:**

CAB+RPV LA is the only complete long-acting regimen for treatment of virologically suppressed people with HIV (PWH). Administered monthly or every 2 months by a healthcare provider (HCP), CAB+RPV LA may alleviate challenges associated with daily oral antiretroviral therapy (ART). Perspectives of PWH receiving CAB+RPV LA in real-world US healthcare settings are needed.

**Methods:**

This 2-year prospective, observational study enrolled treatment experienced PWH following the decision to switch to CAB+RPV LA (monthly or every 2 months) across 30 participating US sites. Participants completed baseline (BL) surveys prior to first injection and follow-up surveys at Month 6 (M6). Surveys assessed challenges with daily oral ART, reasons for initiating CAB+RPV LA, HIV treatment satisfaction using the HIV Treatment Satisfaction Questionnaire (HIVTSQ), preference for daily oral vs. injectable, and benefits of more frequent clinic visits.

**Results:**

A total of 308 PWH were enrolled and completed BL surveys (Table 1); 217 PWH had reached the M6 timepoint and completed M6 surveys as of data cut-off (Jan 2023); of the 217 PWH, 8 reported they had discontinued CAB+RPV LA. At BL, 49% respondents reported sometimes, often, or always hiding their prior oral ART for fear of disclosing HIV status. The common primary reasons PWH chose to start CAB+RPV LA were: tired of taking daily oral ART, wanted a more convenient treatment option, and worried about missing a dose (Table 2). At M6, PWH receiving CAB + RPV LA reported a decrease from BL in fear of disclosure, anxiety around adherence, and daily reminder of HIV. At M6, 88% of PWH reported CAB+RPV LA was rarely or never an unwelcome reminder of their HIV status vs. 50% at BL with prior oral ART. Most participants preferred CAB+RPV LA (95%), 2% preferred daily oral ART, and 2% had no preference at M6. Treatment satisfaction increased from BL to M6; most reported multiple additional benefits with more frequent clinic visits (Table 3).

**Figure:**
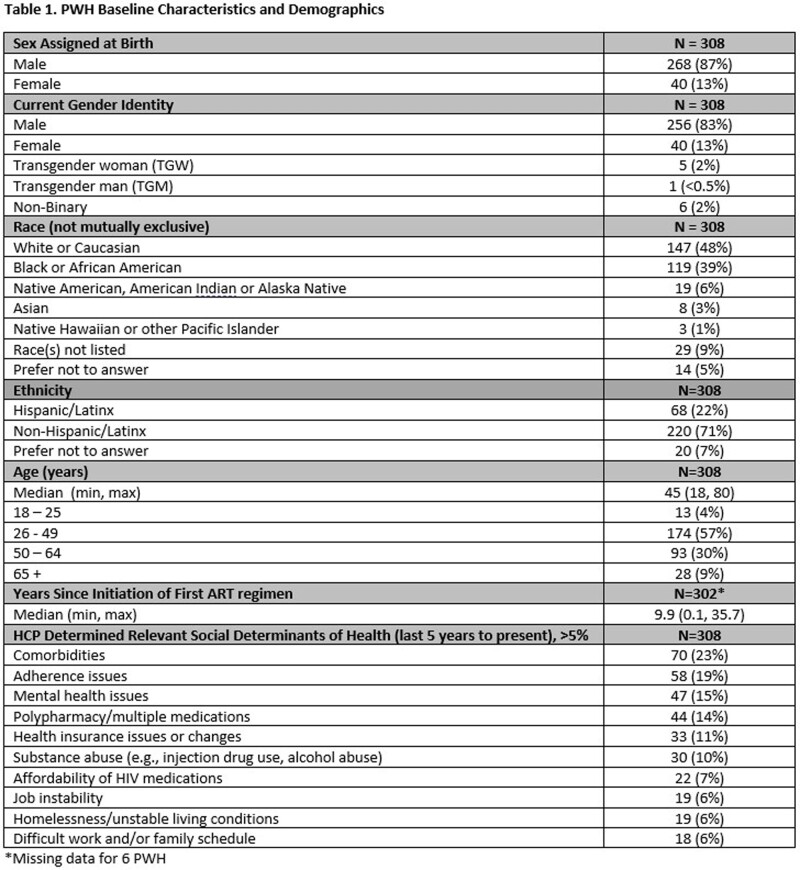

**Figure:**
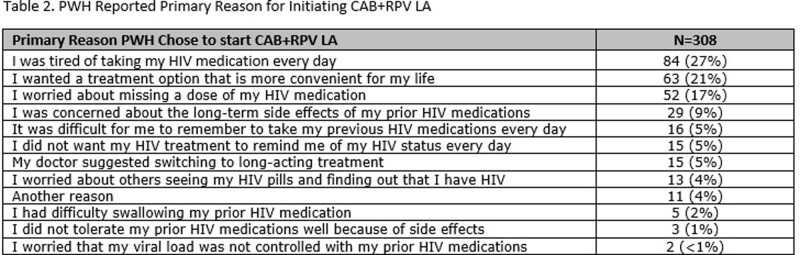

**Figure:**
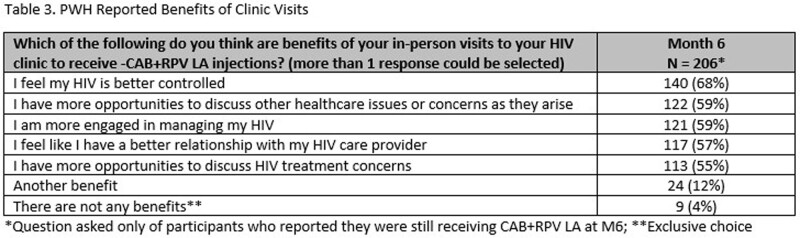

**Conclusion:**

Switching to CAB + RPV LA demonstrated improvements in fear of disclosure, anxiety around adherence, and daily reminder of HIV status at M6 (Fig. 1-3). PWH reported a strong preference for CAB+RPV LA, increased treatment satisfaction, and more opportunities to engage with their HIV care.

**Figure:**
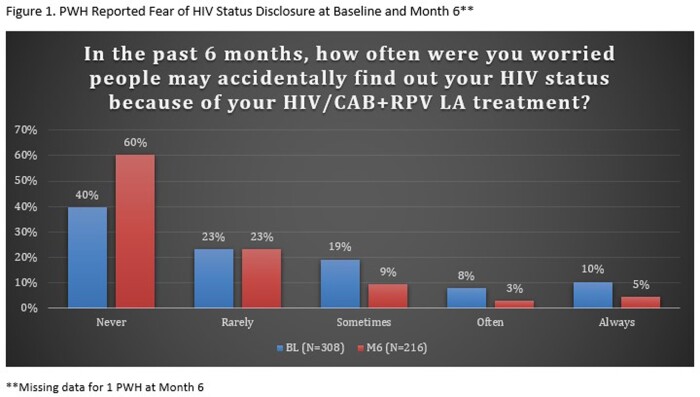

**Figure:**
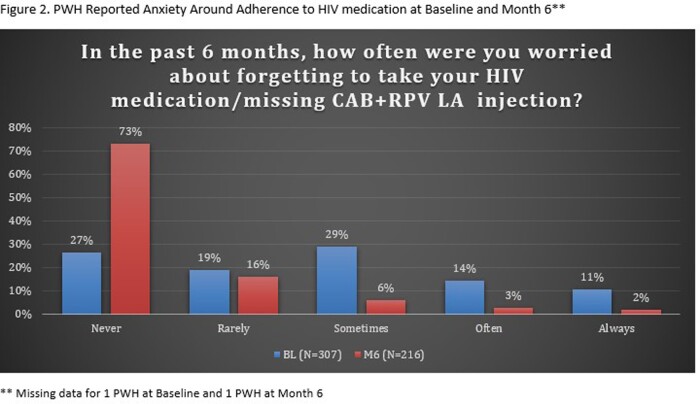

**Figure:**
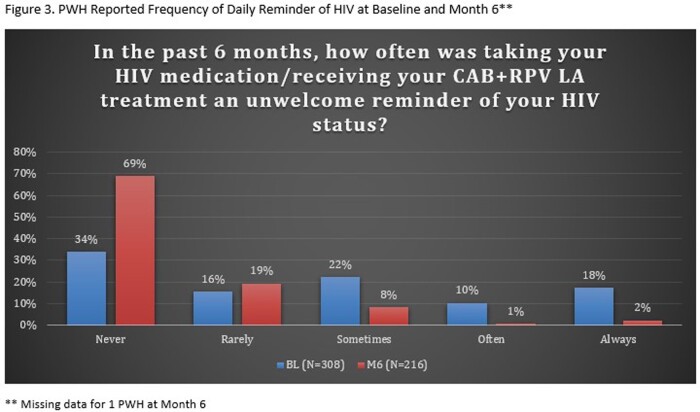

**Disclosures:**

**Dima Dandachi, MD, MPH**, ViiV Healthcare: Advisor/Consultant|ViiV Healthcare: Grant/Research Support **Douglas Cunningham, DO**, ViiV Healthcare: Advisor/Consultant **William M. Valenti, MD, FIDSA**, Gilead: Grant/Research Support|ViiV Healthcare: Grant/Research Support **John Phoenix, MSN, APRN, FNP-C**, Gilead: Grant/Research Support|Gilead: Speaker Bureau|Huntridge Family Clinic: Ownership Interest|Napo Pharmaceuticals: Speaker Bureau|ViiV Healthcare: Grant/Research Support|ViiV Healthcare: Speaker Bureau **Paula Teichner, PharmD**, GlaxoSmithKline: Stocks/Bonds|ViiV Healthcare: Employment **Maria Reynolds, MStat**, RTI Health Solutions: Employment|ViiV Healthcare: Grant/Research Support **Cindy Garris, MS**, GSK: Stocks/Bonds|ViiV Healthcare: Employee

